# Nutritional priorities, practices and preferences of athletes and active individuals in the context of new product development in the sports nutrition sector

**DOI:** 10.3389/fspor.2023.1088979

**Published:** 2023-02-07

**Authors:** Conor C. Carey, Lorna Doyle, Alice Lucey

**Affiliations:** ^1^ Cork Centre for Vitamin D and Nutrition Research, School of Food and Nutritional Sciences, University College Cork, Cork, Ireland; ^2^ Department of Sport and Exercise Science, South East Technological University, Waterford, Ireland

**Keywords:** sports nutrition, new product development, food choice, functional foods, exercise, ergogenic aid, sports foods, supplements

## Abstract

**Introduction:**

Sports nutrition is a rapidly growing sector with increasing demand for evidence-based nutritional products to support competitive and healthy lifestyles. The product development process for novel foods should rely heavily on end-user engagement to facilitate future success, however there is a dearth of published information available. An understanding of the practices and self-reported nutritional priorities of athletes and active individuals is required for the development of new food products, facilitating evidence-based product formulation.

**Methods:**

Participants were at least 18 years of age, actively participating in competitive sport or structured physical activity on at least two occasions per week. Participants were asked to undertake a comprehensive online survey assessing their nutritional practice, perceived nutritional priorities and preferences for product characteristics. Questions were developed on the basis of critical evaluation of the current scientific literature and the hosting of two scoping focus group sessions with prospective end-users.

**Results:**

405 individuals (29 ± 9 years) completed this questionnaire. 295 participants reported active participation in competitive sport while the remaining 110 participants undertook structured physical activity exclusively. When asked to rank their top three most sought-after product claims in sports nutrition, “enhanced muscular recovery” was the most prioritised receiving 101 first choice preferences (25%) and 295 top 3 preferences. Fifty-eight percent of participants reported taking nutritional supplements. Caffeine containing functional foods (excluding caffeine supplements) were the most commonly used functional food group. A very low incidence of functional food usage was reported otherwise. When asked to rank the importance of various food product attributes, “nutritional profile” was ranked as the most important with rating of 3.37 ± 0.7 out of 4 followed by “taste” and “accessibility”. Whole food nutritional products received the most first preference selections and most top 3 selections when presented with a number of popular performance and recovery products on the market.

**Conclusions:**

The transition towards a food first approach in sports nutrition is vital for athletes and active individuals to achieve their goals; with the development of evidence-based functional foods, particularly with a focus on muscle recovery, endurance, and strength enhancement at the forefront for new food product design and innovation.

## 1. Introduction

Sport and exercise nutrition is a rapidly growing sector, with the global market valued at $40 billion USD in 2021 and projected to grow annually by 8.5% between 2022 and 2030 (1). This increasing global demand has been matched with additional availability of evidence-backed nutrition solutions, some of which have been proven to assist athletes with performance, enhance post exercise recovery, and augment body composition (2) The global increase in life expectancy which parallels concomitant increases in the prevalence of chronic disease, has resulted in growing demand for evidence-backed solutions to support population health (3). Given that physical activity (4) and diet and nutrition (5) are two of the most effective and widely used prophylactic approaches for chronic disease prevention, the pragmatic development of food-based solutions not just for athletes, but to support healthy and active lifestyles across the lifespan is imperative. A considerable issue that has plagued the sports nutrition industry has been the simultaneous rise in non-science backed nutrition products arriving into the market with spurious efficacy claims (6). The development of an evidence base to enable and underpin new product development strategy is essential to support the forthcoming growth in this sector and to tackle the challenge of credibility of product claims within this sector.

Advances driven by both the scientific community and food industry have underpinned a clear transition towards personalised (7, 8) and periodised (9) nutritional practices, where a number of products have been shown to be efficacious in randomised controlled trials, when best practice protocols are applied to appropriate population groups (10–13). However, converting these positive findings from highly controlled scientific trials to free living situations is a considerable challenge for this industry. Furthermore, the availability of information about nutritional strategies for these population groups has increased dramatically in recent years related to the rise of internet and social media use, with Bourke et al. (2019) reporting 65% of athletes using social media for nutrition purposes in the previous 12 months (14). While the internet and social media provides end-users with rapid and cost-effective access to nutritional information, the conditions and caveats that arise from personalised and periodised approaches are often difficult to fully comprehend through the communication limitations of social media. This poses notable difficulties for athletes and active individuals with some athletic groups in recent years even exhibiting poorer nutrition knowledge than their community counterparts (15) while others have reported that while general nutrition messages were well understood, notable gaps in nutrition knowledge were present (16). To date much of the literature assessing the perceived nutritional priorities of this population has focused on assessing nutrition knowledge in athletes and their support staff, with a number of studies suggesting a critical need for greater food and nutrition literacy (17–19). It is clear that those with access to qualified nutritional professionals such as sports dietitians are at a considerable advantage on this front (20). Potential reasons for this disparity may be poor access to qualified nutrition professionals among different sporting groups, particularly for those not at elite level and those of lower socioeconomic backgrounds (21–23). It is clear that improving nutrition and health literacy in the athletic population represents a significant opportunity to improve both performance, health and wellbeing (24, 25). Gaining a quantifiable understanding of the current practices is critical to shape future food innovations in the sports nutrition sector, allowing for greater understanding of where gaps in knowledge exist and whether current perceived priorities of this population are misevaluated.

Factors influencing food choice in athletes include culture, physiological demands, and socioeconomic factors amongst others, highlighting the complexity of food choice in sporting populations (26). However, one study showed that within NCAA division I and III athletes, there was no influence of demographic or athletic characteristics on supplement usage (27). Wesana et al. (2020) analysed the brand equity and preference for sports nutrition brands concluding that sporting factors such as competition level and sport type as well as more generic socio-demographic determinants including age, gender and education play a considerable role in brand perception and food choice among consumers (28). The characteristics of the food product itself also have an impact on whether a product will be adopted by the target population in free-living scenarios. It is well established that sensory factors such as taste, aroma, texture and appearance play a crucial role in food choice within the general population (29). While these may not be quite as important in elite athletic populations (26), they may prove to be the deciding factor when two competing sports nutrition products have physiologically equivalent outcomes. A key example of this product optimisation has been the emergence of low volume, high nitrate concentrated beetroot juice shots in place of larger volumes of standard beetroot juice. Despite this however, there is a complete dearth of scientific knowledge investigating athletes and active individuals' preference for sensory characteristics of sports nutrition food products despite their importance for food choice and decision to purchase.

Taking a broad overview of the sports nutrition sector, it is unclear as to the appropriate focus when aiming to design and produce products to bring to the sport and exercise market. Due to the multitude of factors affecting food choice (26), it is largely unclear which prototype may develop into a successful product in the market. Combine this with the reported key nutritional knowledge gaps reported in these populations (15, 16), it is exceptionally difficult for the sports nutrition sector to effectively identify the correct avenue for future new product development ventures in order to develop a scientifically-proven efficacious product that will be adopted by the end-user. This novel cross-sectional research was conducted with an overarching objective to inform key priorities and design elements for future new product development through assessment of current practices, perceived nutritional priorities and product preferences of athletes and active individuals.

## 2. Methods

### 2.1. Research approach

The research tool (a comprehensive online survey questionnaire) underpinning the data presented in this study was developed using a combination of critical review of current scientific evidence, in conjunction with end-user engagement through the conduction of two scoping focus group sessions with athletes and active individuals of mixed backgrounds and abilities. The findings of these focus groups enabled the design of the key questions utilised in the online questionnaire allowing the targeted identification of particular disconnects between current product offerings and the needs of end-users, both known and hidden. Data from these focus group sessions were analysed and did not reach idea saturation while further focus groups sessions were cancelled due to the onset of the covid-19 pandemic. Through critical review of the available literature investigating nutritional knowledge, practices and food choice within sport and exercise, knowledge gaps were identified which are of high value for informing future product development and the creation of this holistic research tool (27–35). Notably, these included the lack of published publicly available data to underpin design elements of new sports nutrition products such as product claim prioritization, the relative importance of nutritional and product characteristics to the end user and consumer preferences for various product forms currently available on the market.

### 2.2. Participants

Participants in this study were at least 18 years of age. The targeted population group for this study was athletes and active individuals which was defined as anyone undertaking competitive sport or actively participating in a form of physical activity program on at least two occasions per week. This was to ensure that participants were at least at a level where diet and nutritional practices would be potentially influential to their exercise or sporting performance and represented the predominant end-user group of sports nutrition products. Participants were recruited through social media advertisement, internal university email list and word of mouth. Clubs, organizational bodies and sporting facilities were also approached to distribute this survey to their athletes and members. A convenience sampling method was used to achieve this sample and the sample size is in line with that of similar survey-based research in nutrition, food choice and sports nutrition (14, 23, 32, 36).

### 2.3. Procedures

This questionnaire was granted ethical approval from the Social Research Ethics Committee of University College Cork (log 2020-046). All responses were provided anonymously, and no identifying data was collected. Prior to participation, participants were presented with the purpose of the questionnaire, their role within the research and were given contact details of the researchers should they have any questions regarding the research project. Following this, participants were required to provide their informed consent to participate.

This questionnaire was carried out using the online platform Qualtrics (Qualtrics, Utah, USA). The questionnaire utilised a series of thirty-four questions including multiple-choice questions, rating scale questions, rank order questions and matrix scale questions. Questions were designed to assess the current priorities, practices and preferences of athletes and active individuals regarding their nutrition and food choices. Questions related to preferences for product characteristics and food types were based of common product offerings in the sports nutrition market following review of various a wide array of sports nutrition product offerings available in Ireland and online.

### 2.4. Data analysis

Data was extracted from Qualtrics for further statistical analysis. All data was analysed statistically using IBM SPSS (IBM SPSS Statistics version 26.0, IBM Corp, NY, USA). Data is described as mean ± standard deviation (±SD), frequencies and percentages where appropriate. Certain questions contained open text fields labelled “other” which were grouped for analysis when possible and analysed quantitatively. All data was initially analysed as an entire dataset, following this, various questions were analysed by subgroup using Mann Whitney U tests. Subgroups assessed were gender, competition level and total activity time per week. Statistical significance was set at *P* < 0.05 for all statistical tests.

## 3. Results

### 3.1. Demographics

Four-hundred and five (164 female, 241 male) athletes and active individuals successfully completed this questionnaire and were eligible for inclusion in analysis. The mean age of respondents was 29 ± 9 years. A total of 295 participants (73%) reported active participation in competitive sport while the remaining 110 participants (27%) undertook a non-competitive structured physical activity programme exclusively. Participants reported undertaking 10.3 ± 6.6 h per week of either sporting or structured physical activity with those involved in competitive sport reporting 8.4 ± 4.9 h of sporting activity or physical preparation for competition per week alongside an additional 4.5 ± 4.0 h of structured physical activity separate from their sporting activity (Table 1).

### 3.2. Food and nutritional priorities of athletes and active individuals

#### 3.2.1. Rank your top 3 product claims when choosing whether to use a performance food or product

**Figure 1:**
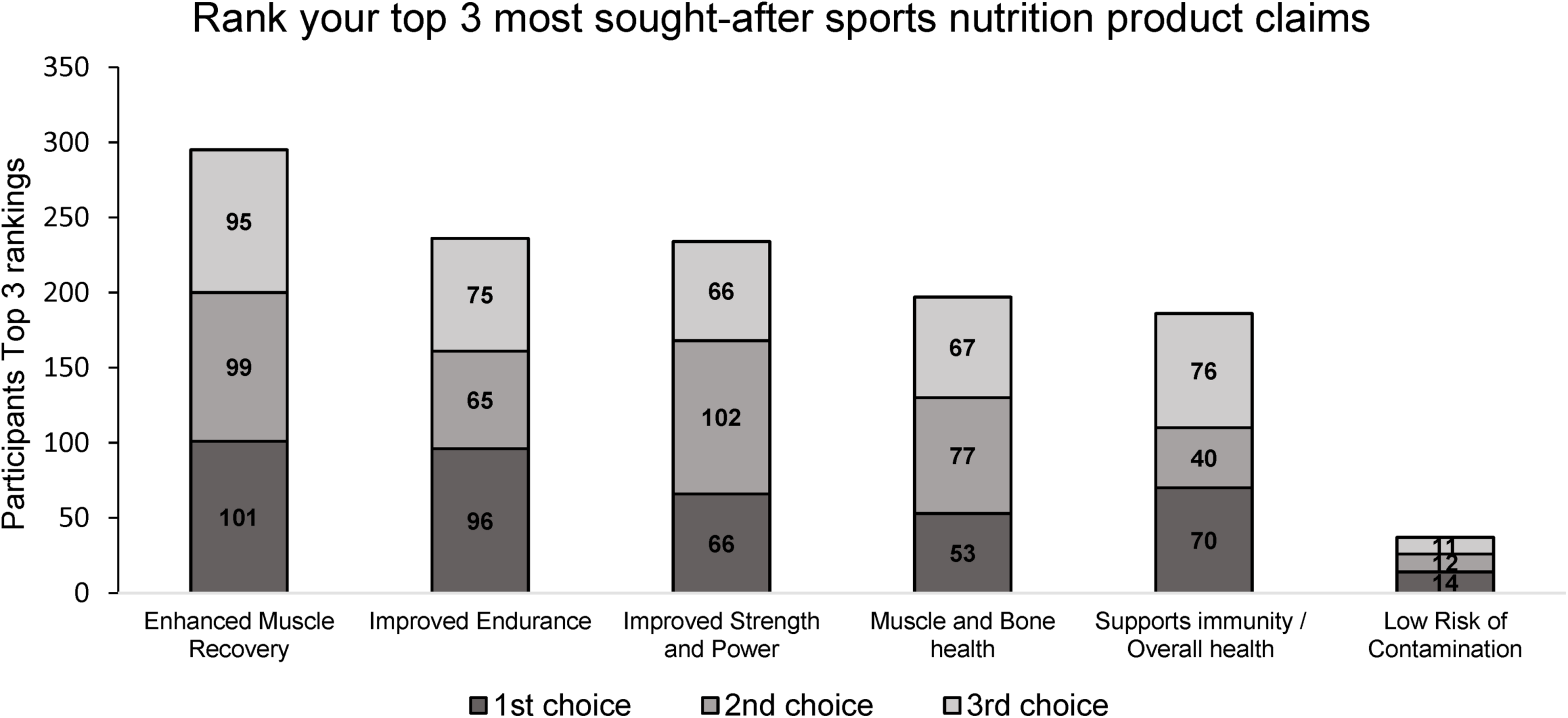
The most sought-after sports nutrition product claims of athletes and active individuals.

When asked to rank their top three most sought-after claims of sports nutrition products, “*enhanced muscular recovery”* received the most first choice preferences at 25% (*n* = 101); while 73% (*n* = 295) participants ranked enhanced muscular recovery in their top 3 priorities overall (Figure 1). “*Improving endurance*” and “*improving strength and power*” were the next most sought-after nutritional product claim with 58% (236) and 57% (234) of participants rating in their top 3 priorities respectively. However, “*improving endurance”* received considerably more top priority selections (24%, 96) compared to “*improving strength and power” (16%, 66)*.

#### 3.2.2. Does your engagement in physical activity frequently cause a stiffness and/or pain in your muscles in the hours and/or days following exercise? If so, how frequently do you experience this stiffness or pain?

Seventy percent of participants reported frequently experiencing muscular pain and stiffness post exercise, with 56% (228) reporting pain or stiffness at least once per week.

### 3.3. Current practices of athletes and active individuals

#### 3.3.1. Do you currently take nutritional supplements or bioactive functional food products?

Fifty eight percent of participants reported currently taking nutritional supplements, with 52% (*n* = 210) reporting supplement use for health and wellbeing purposes and 35% (n = 141) reporting supplement use to support their exercise performance or recovery. Multivitamins were the most commonly reported supplement used for health and wellbeing while protein supplements were the most commonly used exercise-related supplements (Table 2). Forty three percent (*n* = 173) of participants reported using bioactive functional foods as part of their nutritional routine to improve exercise performance or recovery. Caffeine-containing functional foods were the most commonly used group of functional foods with 135 participants reporting ingesting coffee or other caffeine containing food products to support their exercise performance (Table 2).

**Table 1:** Demographics of questionnaire respondents (*n* = 405).

	Mean ± SD (range)	Frequency (%)
**Gender**		
Males		241 (59.5%)
Females		164 (40.5%)
**Age (yrs.)**	29 ± 9 (18 to 64)	
**Total Activity Time**	10.3 ± 6.6 (1 to 51)	
Hours per week		
**Competitive Sport Participation**		
Yes		295 (72.8%)
No		110 (27.2%)
**Sport Category**		
R.I.D.S		192 (58.9%)
Endurance		37 (11.3%)
Combat		27 (8.3%)
Aquatic		23 (7.1%)
Racket		22 (6.7%)
Resistance/power		10 (3.1%)
Dance		5 (1.5%)
Equestrian		3 (0.9%)
Other		7 (2.1%)
**Level of Competition**		
International		31 (10.5%)
National		56 (19.0%)
Regional		73 (24.8%)
Local		133 (45.2%)

RIDS, Random intermittent dynamic type sports e.g. Soccer, Rugby, Basketball.

**Table 2:** Prevalence of supplement and functional food usage in athletes and active individuals (*n* = 405).

What nutritional supplements are you currently taking for health and wellbeing purposes?		What nutritional supplements do you currently take for exercise performance or recovery?		Do you use any “functional food products”, specifically for exercise performance or recovery?	
Multivitamin	114 (28%)	Protein	125 (31%)	Caffeine	135 (33%)
Vitamin C	92 (23%)	Creatine	56 (14%)	Polyphenols	24 (6%)
Vitamin D	79 (20%)	Caffeine	46 (11%)	Dietary nitrate	14 (4%)
Omega-3 PUFA	77 (19%)	BCAA's	28 (7%)		
Iron	50 (12%)	Carbohydrate	8 (2%)		
B Vitamins	50 (12%)	Beta Alanine	4 (1%)		
Calcium	29 (7%)	Glutamine	3 (1%)		
Magnesium	13 (3%)	Citrulline	1 (<1%)		
Folic Acid	10 (2%)	L Carnitine	1 (<1%)		
Zinc	4 (1%)	Arginine	1 (<1%)		
Melatonin	2 (<1%)				
Glucosamine	2 (<1%)				

#### 3.3.2. Do you alter your routine after training/exercise/competition to improve recovery?

**Figure 2:**
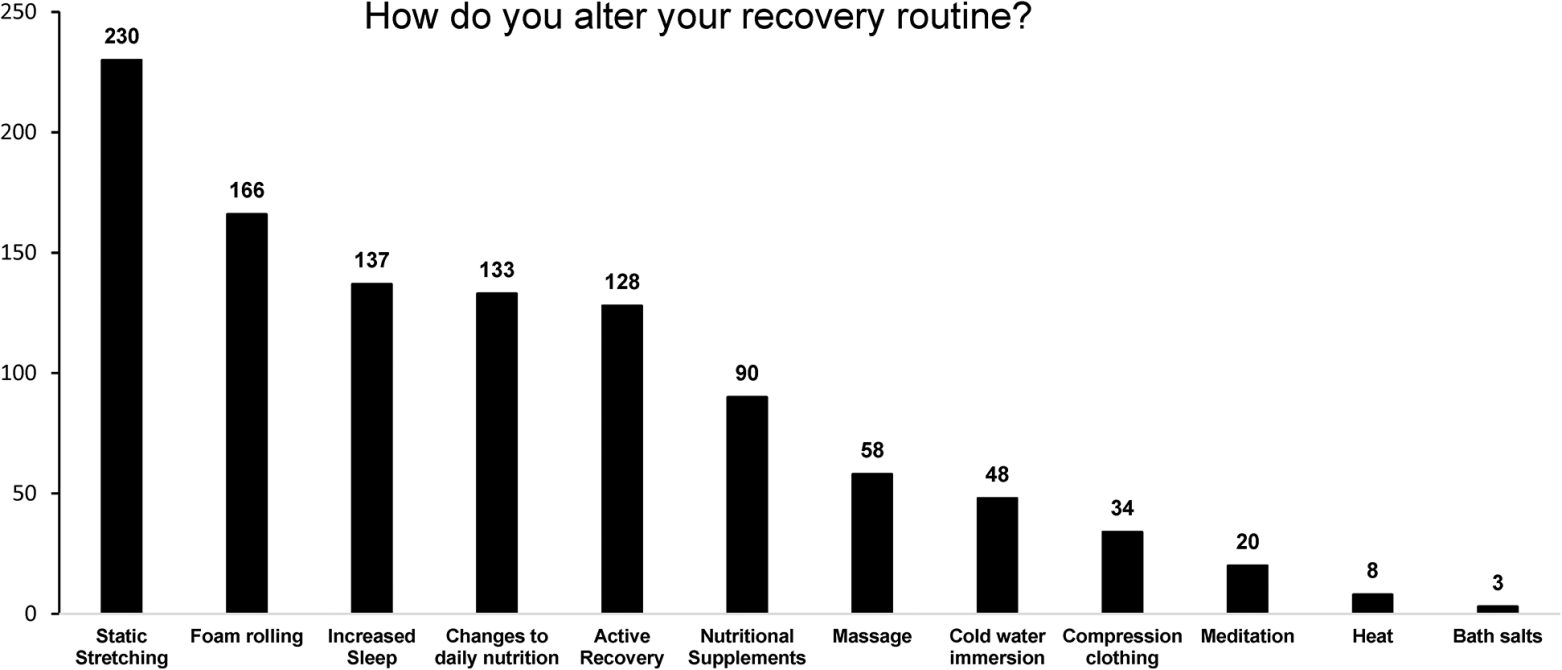
Recovery practices of athletes and active individuals (*n* = 285).

Seventy percent of participants reported altering their routine surrounding exercise with the intention to improve their recovery. Of the 285 participants who alter their routine, the most popular recovery routines were stretching (*n* = 230) and foam rolling (*n* = 166) (Figure 2).

### 3.4. Food and nutritional preferences of athletes and active individuals

#### 3.4.1. On a scale of 1–4 how important would the following features be in your decision to purchase a food for exercise performance or recovery?

When asked to rank the importance of various features of a product for exercise performance or recovery, nutritional profile was ranked as the most important with an average rating of 3.37 ± 0.65 out of 4, followed by taste (3.15 ± 0.80) and ease of access (3.09 ± 0.72) (Table 3). Mann Whitney U analysis indicates that females rated “taste” (U = 14,311, Z = −5.031, *P *< 0.001), “price” (U = 17,311, Z = −2.021, *P *= 0.043), “nutritional profile” (U = 15,537, Z = −3.710, *P *< 0.001), “ease of access” (U = 17,086, Z = −1.989, *P *= 0.047) and “sustainability” (U = 13,607, z = −5.220, *P *< 0.001) as more important than did their male counterparts, while gender had no impact on the “ease of preparation”.

**Table 3:** Importance of product features and scientific proof on nutrition product choice using a scale of 1 (not important) to 4 (crucial). (*n* = 405).

On A Scale of 1–4 how important would the following features be in your decision to purchase a food for performance/recovery:					
	1 (not important)	2	3	4 (crucial)	Mean Score ± SD
Nutritional Profile	<1%	7%	46%	46%	3.37 ± 0.65
Taste	6%	9%	51%	35%	3.15 ± 0.80
Accessibility	2%	16%	53%	29%	3.09 ± 0.72
Ease of Preparation	4%	20%	47%	29%	3.02 ± 0.80
Price	4%	15%	57%	24%	3.01 ± 0.74
Sustainability	16%	37%	34%	14%	2.45 ± 0.92
On a scale of 1–4, how important is it that the effects of a sports nutrition product have been scientifically proven?					
	1 (not important)	2	3	4 (crucial)	Mean Score ± SD
Scientific Proof	<1%	13%	41%	45%	3.31 ± 0.72

Athletes who reported competing at local or regional level rated the “ease of preparation” of a product as significantly more important than did those competing at national or international level (U = 7467, Z = −2.209, *P *= 0.027), however “level of competition” had no impact on the importance of “taste”, “product pricing”, “nutritional profile”, “ease of access” or “sustainability” (*P* > 0.05). Participants reporting less than 10 h per week of total sport or structured physical activity rated the “price” of a sports nutrition product more important than those undertaking greater than 10 h per week (U = 17,004, Z-2.129, *P *= 0.033). Hours of activity per week had no significant impact on importance of “taste”, “nutritional profile”, “ease of preparation”, “ease of access” or “sustainability”.

#### 3.4.2. Rank in order, your top 3 preferred types of recovery product from the following list of common recovery products & rank in order, your top 3 preferred types of performance enhancing nutrition product from the following list of performance products

Whole food type nutritional products received the most first preference selections and most top 3 selections when presented with a number of popular performance and recovery products on the market (Table 4). Whole foods received 186 and 180 first preferences for recovery and performance products, respectively. Food bars received the second most top 3 preferences in both recovery and performance categories with 68% (*n* = 274) and 65% (*n* = 262) participants ranking food bar products in their top 3 preferences respectively. Powder-based products received the second most number one preferences in recovery and performance categories with 56 top preference selections in the recovery category and 58 top preference selections in the performance category. When presented with a list of common food products which often carry performance or recovery claims, a smoothie or juice option received 202 top three rankings, however a hot food option received the most top rankings with 101 participants responding that it was their favourite of the food options presented.

**Table 4:** Descriptive data outlining the sports nutrition food product formats preferences of athletes and active individuals (*n *= 405).

Rank in order, your top 3 preferred types of recovery enhancing nutrition product.												
	1st Choice			2nd Choice			3rd Choice			Total Top 3 selections		
	Males	Females	Total	Males	Females	Total	Males	Females	Total	Males	Females	Total
Whole Food	114	72	186	49	38	87	31	20	51	194	130	324
Bar	24	26	50	44	50	94	81	49	130	149	125	274
Dairy	23	31	54	52	33	85	46	40	86	121	104	225
Powder	46	10	56	61	17	78	34	20	54	141	47	188
Pre-made Beverage	27	14	41	24	14	38	27	17	44	78	45	123
Pill/Capsule	3	7	10	8	7	15	14	9	23	25	23	48
Rank in order, your top 3 preferred types of performance enhancing nutrition product.												
	1st Choice			2nd Choice			3rd Choice			Total Top 3 selections		
	Males	Females	Total	Males	Females	Total	Males	Females	Total	Males	Females	Total
Whole Food	96	84	180	32	13	45	35	24	59	163	121	284
Bar	28	22	50	53	62	115	61	36	97	142	120	262
Pre-made Beverage	31	17	48	37	26	63	41	30	71	109	73	182
Powder	42	16	58	50	17	67	21	25	46	113	58	171
Gel	32	8	40	39	17	56	36	17	53	107	42	149
Pill/Capsule	5	8	13	14	10	24	17	7	24	36	25	61
Gum	2	2	4	5	4	9	11	9	20	18	15	33
Rank in order, your top 3 preferred form of sports nutrition food product.												
	1st Choice			2nd Choice			3rd Choice			Total Top 3 selections		
	Males	Females	Total	Males	Females	Total	Males	Females	Total	Males	Females	Total
Smoothie/Juice	36	38	74	52	28	80	41	25	66	129	91	220
Shake	80	19	99	38	11	49	29	18	47	147	48	195
Bar/Flapjack	19	21	40	37	33	70	46	28	74	102	82	184
Hot Food	70	31	101	24	9	33	27	12	39	121	52	173
Yoghurt	5	12	17	14	16	30	24	15	39	43	43	86
Pancake/Brownie	5	9	14	17	13	30	19	18	37	41	40	81
Spreadable	4	18	22	12	25	37	6	12	18	22	55	77
Cereal	10	1	11	23	11	34	10	9	19	43	21	64
Biscuit/Cookie	3	3	6	6	5	11	15	7	22	24	15	39
Hot beverage	2	1	3	8	0	8	10	6	16	20	7	27
Shot	3	4	7	4	3	7	7	4	11	14	11	25

## 4. Discussion

### 4.1. Overview of study findings

While there has been substantial research advancement in efficacy testing of potential product prototypes (37); there is often considerable difficulty when it comes to translation of theoretically efficacious product prototypes to successful adoption amongst consumers (38). In order to achieve this, an evidence-based needs analysis of target market must be evaluated to inform the conversion from food prototype to successful product.

The data gathered from this study has identified a clear disconnect between certain aspects of current practice of this population and advised evidence-based best-practice. Key findings from this study clearly demonstrate the demand for effective sports nutrition solutions to provide support for post exercise recovery, with 25% of participants highlighting this as their number one priority when it comes to their nutritional practice. While effective sports nutrition solutions to provide support to post exercise recovery are deemed of critical importance to these end-users; we detected that there is a clear disconnect between the current practice of end-users and the recommended scientific best-practice within the topic. There is also undoubtedly a desire for more food-based solutions to be developed, yet it is clear from current practice that supplementation still represents a considerable majority of the sports nutrition products produced and consumed (39). Future ventures should prioritise the development of fortified and functional food alternatives as evidenced in findings of this research study.

### 4.2. Priorities of athletes and active individuals when choosing a sports nutrition product

It is critical to gain a quantifiable understanding of what aspects of physiological function this population prioritise as being important to support both their health and athletic performance. To the best of the authors knowledge this is the first study to analyse the specific product claims and attributes which athletes and active individuals prioritise when it comes to selecting sports nutrition products.

#### 4.2.1. Post-Exercise recovery

This study demonstrates for the first time that post-exercise recovery is the most sought-after sport and exercise nutrition product claim with almost 3 in 4 participants ranking muscular recovery as one of their top three priorities, and one quarter ranking it as their top priority. Exercise-induced muscle damage (EIMD) is wide ranging in its prevalence with 7 in 10 participants reporting frequently experiencing muscle stiffness or pain post exercise. EIMD is caused by unaccustomed strenuous exercise particularly when such exercise is at high intensity or contains high eccentric loading (40, 41). Over half of participants, reported experiencing stiffness or pain caused by EIMD at least once per week, highlighting the magnitude of this issue and the urgent need for an evidence-based food solution. Using appropriate methods to recover from EIMD allows athletes and active individuals to achieve the greatest possible adaptation to strenuous exercise through allowing for increased training frequency and also reduced the time spent in a state of compromised muscle function (42). To enhance recovery after exercise, evidence suggests that protein supports muscle adaptation, and polyphenol-rich foods like tart cherry juice can be effective nutritional strategies to improve recovery from muscle soreness and damage (42, 43).

#### 4.2.2. Improving strength and endurance

Improving both endurance and strength through the use of nutritional products followed closely as important product claims prioritised by this population ranking them second and third respectively in terms of product claim importance The emergence of food forms such as isotonic sports drinks, gels and shots have attempted been developed to provide in-competition carbohydrate fuelling options, further innovation is duly warranted in this area. While protein ingestion coupled with resistance training improves strength and power adaptation in the long term (44, 45); improving acute strength and power performance through nutritional means is a decidedly more difficult challenge. Caffeine shows considerable efficacy in this regard (46), and also in improving acute endurance performance (47), however issues relating to dosages, habituation and genetic variance in response (48) mean that achieving an optimal ergogenic effect may prove challenging for the athlete. Creatine monohydrate supplementation also provides potential for an ergogenic effect, increasing short term strength and power performance (49, 50), however doses, particularly during loading phases appear achievable *via* supplementation and not by dietary means (49).

#### 4.2.3. Health and immunity

Despite this research being carried out during the covid-19 pandemic which likely placed greater emphasis on maintaining health and immunity, this product claim was less prioritised in comparison with muscle recovery, enhancing strength and enhancing endurance. This strongly suggests that when it comes to nutrition and dietary interventions this population of athletes and active individuals prioritise seeking products that can have a direct impact on sporting performance rather than products that help maintain health and wellbeing exclusively. Given the considerable risk of absence from training and competition associated with illness and infection surrounding major sporting competition, this poses a considerable under prioritisation within this population (51, 52).

#### 4.2.4. Scientific efficacy

Due to the lack of regulation of the sports nutrition market, along with the recent growth in the industry, a considerable level of scepticism over the use of sports nutrition products has emerged in recent years (53, 54). Particularly, the prevalence of mislabelling and contamination of sports supplements has led to a notable movement away from advocating for the use of sports supplements and towards a food first approach to sports nutrition (55). Within the wider food industry there has been considerable improvement within regulation of health claims of food products in recent years with the European Food Safety Authority and the European Commission introducing regulations on Nutrition and Health claims in 2007 [Regulation (EC) No. 1924/2006]. Establishing a minimum standard of scientific evidence underpinning these claims through regulation is a priority (56). This is now extending into the sports nutrition sector with the development of the Australian Institute of Sport ABCD classification of sports foods and supplements (Australian Institute of Sport 2018) and the International Olympic Council releasing their consensus statement on dietary supplements and their claims (57). In the current research, 87% of all participants rated the importance of having scientific proof to support a product claim at least a 3 out of 4 in terms of importance. Furthermore, 84% of participants stated they would be willing to pay extra for a sports nutrition product with a product claim that is scientifically proven. It has been shown previously that scientific evidence backed health claims influence overall perception, food choice and willingness to pay (58) This finding highlights the importance of a rigorous scientific process in new product development practice for sports nutrition food products, and also emphasises the importance of regulating sports nutrition efficacy claims to protect the consumer from spurious or fraudulent efficacy statements. Even when sports nutrition products have well established science-backed efficacy claims, risk of inadvertent product contamination is another particular issue within the sports nutrition industry. Paired with regulation of product claims, recommendation of third-party testing of sports nutrition products and their batches to ensure products contain the stated ingredients only and in the stated dosages is essential for the safety of end-users. Along with this education of end-users around the risks of supplement contamination and the importance of third-party testing is essential for the future of product regulation (59).

### 4.3. Current practices of athletes and active individuals

#### 4.3.1. Supplementation

Supplementation type products represent a majority of the market share for sports nutrition products (1). In our sample ∼60% of participants reported using nutritional supplements, with protein supplements being the most common exercise-related supplement and multivitamins being the most used health-related supplement. These findings closely align with those reported in the meta-analysis of Knapik et al. (2016), who estimated prevalence of the use of any dietary supplement in combined athletic groups at 58% (39). Supplement usage in the general adult population in Ireland, as reported by the National Adult Nutrition Survey, is considerably lower at an estimated 28% highlighting that athletic groups are highly motivated and concerned with adapting their nutritional practices for either health or performance purposes (60). One notable distinction between the results of this study and those presented in Knapik et al. (2016), is the greater prevalence of vitamin D supplementation with 20% of the sample in the current study reporting current supplementation compared to a supplementation prevalence estimate of 7% in the meta-analysis (39). This data was collected in Ireland which has an increased prevalence of vitamin D insufficiency and deficiency (61). This study was conducted during the covid-19 pandemic during which increased focus was placed on vitamin D supplementation to support immune health (62). The prevalence of vitamin D supplementation in Irish athletes and active individuals was higher (20%) than that reported in the general Irish population, at 17.5% (63). Given the growing knowledge surrounding the importance of avoiding vitamin D deficiency in athletes this is likely a positive development (64).

#### 4.3.2. Recovery

When participants reported the methods employed to improve recovery post-exercise the most commonly used methods were static stretching and foam rolling. Research into the effects of static stretching on recovery from EIMD have shown little to no effect on recovery of muscle strength or muscle soreness (65), while foam rolling has been shown to have little effect, other than recovery of range of motion (66, 67). Despite the relative importance to the individual, the management of recovery post exercise appears to be largely misunderstood. Promotion of good sleep hygiene (68) and the incorporation of appropriate changes to daily nutrition practices (42) during periods of intense exercise should be prioritised for the improvement of recovery over practices such as static stretching and foam rolling.

Our research indicates that protein supplementation was the most employed supplementation strategy relating to sporting performance or recovery in this population. While increased protein intake has been shown to positively impact muscle protein turnover and as a result augment the regeneration of muscle tissue post exercise and promote optimal muscle and strength gains particularly during resistance training (69–71), it remains unclear as to whether protein supplementation improves the time course of skeletal muscle recovery. A systematic review and meta-analysis showed little effect of protein supplements on recovery from symptoms of EIMD including muscle strength and muscle soreness (72). Another meta-analysis showed that whey protein supplementation had a small to medium temporal ergogenic effect on recovery of muscle function post resistance exercise training, however less than half of the included studies reported a beneficial overall effect (73). Although increasing protein intake will undoubtedly enhance adaptation to resistance training for most individuals, given this evidence, it cannot be relied upon as a primary method to curb the issue of post exercise discomfort and reductions in performance capability in the aftermath of intense exercise. Alternative solutions should be sought to enhance recovery from EIMD such as those discussed in the key review of this topic by Harty et al. (2019) (43).

#### 4.3.3. Bioactive functional foods

A particularly underutilised avenue in the sports nutrition sector appears to be that of bioactive functional foods which provide physiological benefit beyond that of their macro or micronutrient content. Although there is a strong uptake in the use of caffeine-based functional foods, particularly coffee, the majority of participants in this study reported not using such functional foods at all. Only 24 participants reported using polyphenol-based functional food products such as tart cherry products, green tea and dark chocolate, and 14 participants reported the use of dietary nitrate based functional foods such as beetroot juice. While underutilised, the use of polyphenols for sporting performance (74), recovery of muscle soreness and muscle strength (75) as well as providing health benefits (76, 77) has been the focus of recent research with much of the research showing performance, recovery and health benefits, although effects may be small and precise dosage required requires further investigation. Given the myriad of food sources naturally rich in polyphenols (78), and their demonstratable capacity to address key priorities of athletes and active individuals, there appears significant scope for development of polyphenol rich functional foods for the sports nutrition market. The efficacy for the use of dietary nitrate functional food products, particularly beetroot juice is also well established with meta-analyses showing clear benefits in endurance capacity (79, 80). In this survey, while almost 1-in-4 participants reported that improving endurance performance is their most sought-after attribute of a sports nutrition product and over half of the participants ranked it in their top 3, but only 14 (4%) participants reported using a dietary nitrate product. The emergence of functional foods has been a notable trend in the wider food industry in recent years and it is clear there is strong potential for this to extend to the sport and exercise nutrition sector, however challenges translating research to engaging strategies to support consumer uptake must be addressed (81).

### 4.4. Product preferences of athletes and active individuals

#### 4.4.1. Factors affecting food choice

Nutritional profile was voted as the most important factor affecting a purchasing decision of a sports nutrition product, followed closely by taste. Although there is a fast-growing interest in sustainability in the wider food systems, the sustainability of a sports nutrition product received the lowest mean rating of features presented in this question. This suggests that athletes and active individuals are unlikely to be willing to compromise on other factors in favour of having an improved environmental impact, especially with regards nutritional profile and taste. However, product sustainability still has some importance to this population and may be a viable selling point of a product once other key factors are intact (37).

Gender appears to be a particularly important demographic influencer in this population with gender having a significant influence on the rank importance of taste, price, nutritional profile, ease of access and sustainability, which have been previously shown to influence the adoption of functional foods in the diet (36). Outside of gender, competition level and time spent undertaking sport or structured physical activity may also be a factor in the food preferences of participants, particularly in the aspects of taste, ease of preparation and price (26, 29, 36).

#### 4.4.2. Towards a whole food approach

Food choice in athletes is heavily influenced by the demands of the sport or exercise they are participating in, as well as the timing surrounding the exercise event (26). As a result of this, separate questions were asked as to the types of sports nutrition product they would prefer for either performance or recovery. The most desirable food product type highlighted in both questions was “whole food” type nutrition products. A food first approach has been widely advocated for by sporting bodies as well as in three notable expert consensus statements on sports nutrition (57, 82, 83). There is also clearly considerable demand for sports nutrition food products given the results of the product preference section of this study (Table 4). This food-first approach is widely promoted by nutrition professionals over supplementation-orientated approaches owing to the importance of “food synergy” and the interdependence of food constituents to derive the greatest benefit to human physiology, especially with regards to nutrient digestion and absorption (84). This approach has been shown to be particularly beneficial when it comes to protein intake and muscle protein synthesis and the resulting remodelling of muscle tissue as a result of exercise (85). While protein supplements have shown significant benefits for athletes and healthy ageing populations (69, 71, 86), and research using protein supplements has been integral to the development of protein intake guidelines for these populations, whole protein foods have been suggested to have greater beneficial impact than that of their constituent amino acid content alone (85).

Despite consumer demand and the scientific support for food-first approaches to sport and exercise nutrition, market insights note that 83.6% of the market share of this sector in 2019 was held by sports supplements (1). There is evidently major potential for a significant market swing towards foods for sport and exercise in the coming years. Although a food-first approach should be the first option for nutritional practice in sport and exercise, there is potential to include supplementation to augment this practice, particularly for nutrients which are difficult to consume in sufficient quantities from dietary sources to gain an ergogenic benefit. As such, the recently proposed “*food first but not always food only”* framework may represent the most impactful approach (55). This approach posits that athletes should adopt a food-first approach unless faced with one of six pre-defined scenarios which suggest supplementation may provide additional benefits (55). Future innovations in the sports nutrition market should reflect this and prioritise whole food products where possible, reserving supplementation approaches predominantly for nutrients in which it is impossible or wholly impractical to achieve exclusively from diet.

#### 4.3.3. Opportunities for new product design

To date there is no previously published research to the authors knowledge that addresses end-user desires for particular product forms. As previously discussed general food preference factors such as taste are of great importance to this population and as such creating products which meet the desired specifications are crucial for success in the sports nutrition market (Table 3) (26). In this online survey participants were asked to rank their most preferable food products, when provided with a list of food product types found commonly on the sports nutrition market. Products such as smoothie/juice received the greatest number of top 3 selections, however hot food received the greatest number of first preference choices followed closely by a shake type beverage. This aligns considerably with the move towards a food first approach to sports nutrition as discussed above (55, 85) Given the fruit and vegetable derived nature of underutilised bioactive compounds such as polyphenols and dietary nitrates, smoothies and juices represent a particularly interesting direction for future development with juices such as beetroot juice and tart cherry juice showing particular scientific evidence (87, 88). The creation of convenient and accessible hot meal solutions such as recipes and meal preparation methods for hot food, which meet the macronutrient nutritional demands of this population also appear to be in particular demand. Bar/flapjack type products also performed quite well receiving the third highest number of top three preferences. Participants were also asked to rank their most likely place of purchase for a sports nutrition product with supermarkets being ranked the most likely place of purchase for such products (Table 5). Developing food products which combine appealing sensory factors with favourable nutritional profile could revolutionise the sports nutrition sector from a supplement focussed one, to that of a food industry.

**Table 5:** Descriptive data outlining preferences for place of purchase of a sports nutrition product.

Rank your top 3 in order of likelihood, if you were to purchase a sports nutrition product, from where would you be most likely to purchase it?												
	1st Choice			2nd Choice			3rd Choice			Total Top 3 selections		
	Males	Females	Total	Males	Females	Total	Males	Females	Total	Males	Females	Total
Supermarket	95	68	163	35	33	68	61	33	94	191	134	325
Health Food Shop	38	48	86	80	41	121	48	47	95	166	136	302
Online	87	29	116	49	26	75	44	25	69	180	80	260
Pharmacy	11	16	27	45	48	93	56	34	90	112	98	210
Café/Restaurant	1	0	1	19	10	29	12	15	27	32	25	57
Sport Shop	2	1	3	3	0	3	1	1	2	6	2	8
Gym	1	0	1	0	1	1	1	1	2	2	2	4

### 4.5. Limitations

It is worth noting that this study was undertaken in Ireland so the results may not be fully generalisable to that of the wider athletic population. The sporting activities of this sample, contains a considerable proportion of participants reporting engagement in random intermittent dynamic type sports such as soccer, rugby, Gaelic games and basketball which may not be representative of the sporting populations in certain areas of the world. As a result of the convenience sampling nature of this sample it may not be fully representative of views on a population level and it is impossible to assess whether there would be a notable difference between responders and non-responders to the survey. Due to the nature of the format of the rank order questions, it was not possible to statistically compare answers against different population groups such as across gender and competition level, further research should be considered to elucidate trends of these topics across population sectors and among specific sporting sectors.

## 5. Conclusion

There has been both significant growth in the sports nutrition sector as well as significant progression in the scientific knowledge surrounding nutritional practices to support sport and exercise in recent years. However, at this pivotal juncture for the sector it appears that by listening to the end user, greater efficiency and efficacy can be gained in the new product development process. In fields such as skeletal muscle recovery there are clear disparities between the current practice of athletes and active individuals and the scientific evidence of best practice. A transition towards a food first approach in sports nutrition is vital for athletes and active individuals to achieve their goals, with the development of functional foods, particularly with the focus of muscle recovery, endurance, and strength enhancement at the forefront. This population has also shown considerable support for the scientific process in developing such products and testing their respective efficacy. There appears to be particular enthusiasm towards beverages such as smoothies, juices and shakes as well as food products in bar or hot food format. This research merits consideration and priority in future new product developments in the sport and exercise nutrition sector.

## Data Availability

The raw data supporting the conclusions of this article will be made available by the authors, without undue reservation.
